# Comparison of non-schistosomal colorectal cancer and schistosomal colorectal cancer

**DOI:** 10.1186/s12957-020-01925-5

**Published:** 2020-07-01

**Authors:** Weixia Wang, Kui Lu, Limei Wang, Hongyan Jing, Weiyu Pan, Sinian Huang, Yanchao Xu, Dacheng Bu, Meihong Cheng, Jing Liu, Jican Liu, Weidong Shen, Yingyi Zhang, Junxia Yao, Ting Zhu

**Affiliations:** Department of Pathology, Qingpu Branch of Zhongshan Hospital Affiliated to Fudan University, Qingpu District Central Hospital, No.1158 Gongyuan East Road, Qingpu District, Shanghai, 201700 People’s Republic of China

**Keywords:** Colorectal cancer, Schistosomiasis, Overall survival, Prognosis

## Abstract

**Aim:**

The purpose of this study was to compare clinicopathological features of patients with non-schistosomal and schistosomal colorectal cancer to explore the effect of schistosomiasis on colorectal cancer (CRC) patients’ clinical outcomes.

**Methods:**

Three hundred fifty-one cases of CRC were retrospectively analyzed in this study. Survival curves were constructed by using the Kaplan-Meier (K-M) method. Univariate and multivariate Cox proportional hazard regression models were performed to identify associations with outcome variables.

**Results:**

Colorectal cancer patients with schistosomiasis (CRC-S) were significantly older (*P* < 0.001) than the patients without schistosomiasis (CRC-NS). However, there were no significant differences between CRC-S and CRC-NS patients in other clinicopathological features. Schistosomiasis was associated with adverse overall survival (OS) upon K-M analysis (*P* = 0.0277). By univariate and multivariate analysis, gender (*P* = 0.003), TNM stage (*P* < 0.001), schistosomiasis (*P* = 0.025), lymphovascular invasion (*P* = 0.030), and lymph nodes positive for CRC (*P* < 0.001) were all independent predictors in the whole cohort. When patients were stratified according to clinical stage and lymph node metastasis state, schistosomiasis was also an independent predictor in patients with stage III–IV tumors and in patients with lymph node metastasis, but not in patients with stage I–II tumors and in patients without lymph node metastasis.

**Conclusion:**

Schistosomiasis was significantly correlated with OS, and it was an independent prognostic factor for OS in the whole cohort. When patients were stratified according to clinical stage and lymph node metastasis state, schistosomiasis was still an independently unfavorable prognosis factor for OS in patients with stage III–IV tumors or patients with lymph node metastasis.

## Introduction

Growing pieces of evidence have emerged in recent decades that inflammation is the root of many malignant tumors [1, 2]. As the fourth most common cancer and the second leading cause of cancer deaths in the world [3], CRC represents a growing number of cancers that correlated with inflammatio n[1, 4, 5]. *Schistosoma japonicum* (*S. japonicum*), which is common in Southeast Asia [6], is regarded as a risk factor of CRC development [7]. Schistosomal infestation has been implicated in the etiology of several human malignancies including bladder, liver, and CRC [8, 9]. The prevalent view is that the sequestered eggs in the mucosa and submucosa incite a severe inflammatory reaction with cellular infiltration and consequent granuloma formation. This in turn leads to mucosal ulceration, microabscess formation, polyposis, and neoplastic transformation [10]. But the causal relationship between *S. japonicum* and CRC still remained controversial [11]. Some case reports and descriptive studies from Africa and the Middle East raised the possibility of an association between *S. japonicum* infestation and induction of CRC [12–14]. Nonetheless, the pathological evidence supporting this conclusion is rather weak, while some research demonstrated that *S. japonicum* infestation was unrelated with CRC [15].

In the 1950s, schistosomiasis was epidemic at a large scale in regions along the Yangtze River and in more than 400 counties in South China [16]. Because of the effective prevention and cure measures taken in China in recent years, schistosomiasis has been eliminated in most epidemic regions. However, its spread is not yet completely controlled and schistosomiasis occurs every year in a small number of people in the epidemic regions of China [17]. The Qingpu District of Shanghai used to be one of the 10 areas with serious schistosomiasis epidemic in China [18], and problems of treatment and outcome of a large number of late schistosomiasis patients left over from history are still remaining. Therefore, detailed knowledge about schistosomiasis is necessary to improve the accuracy of clinical prognosis prediction and will shed light on improving our ability to the prevention and control of schistosomiasis.

In the present study, we made a retrospective analysis of schistosomiasis and clinicopathological characteristics in 137 CRC-S patients and 214 CRC-NS patients to investigate the effect of schistosomiasis on CRC patients’ clinical outcomes.

## Materials and methods

### Patients and samples

A total of 351 CRC patients were enrolled in this retrospective study. All patients had undergone primary surgical resection at Qingpu Branch of Zhongshan Hospital affiliated to Fudan University, from January 2008 to August 2016. All of the operations followed the principle: adequate resection margins, en bloc high ligation of the inferior mesenteric artery (IMA), and lymphadenectomy. All circumferential margins were cleared. The number of positive lymph nodes and total number of retrieved lymph nodes were recorded. The inpatient medical records and pathological reports were reviewed, and the patients were followed up by telephone. OS is defined as the interval from the surgical operation date to the last follow-up or death caused by CRC. Inclusion criteria included the following: (i) patients with CRC as primary focus, (ii) none of these patients had received any prior anti-tumor therapy, and (iii) patients were diagnosed as adenocarcinoma by pathology after resection of CRC. Exclusion criteria included the following: (i) Tis tumors, (ii) patients who lacked complete information, (iii) patients with synchronous malignancy, and (iv) patients with survival time less than 1 month.

Two expert pathologists reviewed HE-stained slides to determine the diagnosis and to restage the tumors according to the eighth edition of American Joint Committee on Cancer (AJCC).

### Detection of schistosome ova and assessment of tumor budding

Schistosome ova were observed in all of original HE-stained formalin-fixed paraffin-embedded (FFPE) sections (usually 4–6 slides), which were examined at × 10 and × 40 magnification fields using a conventional light microscope by two pathologists who were blinded to the clinical data. The diagnosis of schistosomiasis was done by finding schistosome eggs in HE-stained slides.

Tumor budding was defined as the presence of de-differentiated single cells or small clusters of up to 5 cells at the invasive front of CRC [19]. To assess tumor budding in the 10-HPF method [20], the invasive front is first scanned at low magnification (× 4 to × 10) to identify areas of highest budding density. Tumor buds are then counted under high magnification (× 40), and the tumor budding count is reported. The evaluation of tumor budding was conducted by two pathologists who were blinded to the clinical data. Five tumor budding counts were used as breakthrough point. In brief, tumor bud counts greater than or equal to 5 were defined as the high group, otherwise as the low group.

### Statistical analysis

The association between schistosomiasis and clinicopathological characteristics was evaluated by using the chi-square and Fisher’s exact tests. The Kaplan-Meier (K-M) curves with log-rank tests were used to determine the prognostic significance for OS. Univariate and multivariate regression analyses were used to identify independent prognostic factors, and *P* < 0.05 was defined as the criterion for variable deletion when performing backward stepwise selection. Statistical analyses were performed using SPSS 22.0 (SPSS Inc., Chicago, IL).

## Results

### Clinical characteristics in full cohort

**Fig. 1:**
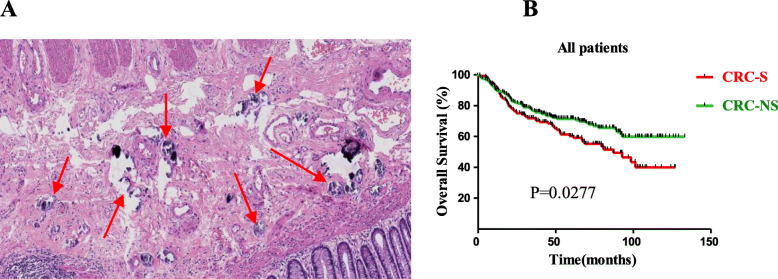
**a** Typical sample of schistosomiasis-associated CRC; the red arrows indicate schistosome ova (HE, × 100). **b** K-M analysis of OS in the whole CRC cohort according to schistosoma infection status. *P* value was calculated by log-rank test

A total of 351 surgically resected FFPE primary CRC samples were included in the study. In the whole cohort, 39.0% (137 out of 351) were infected with schistosoma (Fig. 1a). The clinical and pathological features of the cohort are summarized in Table 1. In the whole cohort, the age of patients at diagnosis ranged from 33 to 91 years (median, 69 years) and they were predominantly male (60.2%, 212 out of 351). By anatomic site, 27% tumors were in the rectum, 33% in the left colon, and 40% in the right colon. Lymph node metastasis was observed in 40% of patients, and 46% of patients were at late-stage disease, while patients without lymph node metastasis were 60%. On the basis of the AJCC Staging Manual (seventh edition), there were very few highly differentiated cases in the follow-up data. Thus, highly differentiated and moderately differentiated cases were classified as “well differentiation,” and poorly differentiated cases classified as “poor differentiation.” Seventy-six percent cases were well differentiated, and 24% were poorly differentiated. As shown in Table 1, lymphovascular invasion, perineura invasion, lymph nodes positive for CRC, and tumor budding were prone to appear in patients with stage III–IV tumors or patients with lymph node metastasis. More poorly differentiated tumors and deeper tumor invasion depth were also mostly observed in patients with late tumor stage or patients with lymph node metastasis. The distribution trend of other clinicopathological features, such as colonic perforation, ulceration, and histological type, was similar within different subgroups.

**Table 1:** Clinicopathological characteristics of the CRC cohort

Characteristics	All patients (*N* = 351)		Patients with stage I–II disease (*N* = 192)		Patients with stage III–IV disease (*N* = 159)		Patients with LNM (*N* = 144)		Patients without LNM (*N* = 207)	
	*N*	%	*N*	%	*N*	%	*N*	%	*N*	%
**Age (< 60 years)**	83	*24*	46	*24*	37	*23*	34	*24*	49	*23*
**Gender (male)**	214	*61*	118	*61*	65	*59*	71	*49*	123	*57*
**Tumor location**										
Rectum	94	*27*	50	*26*	44	*28*	37	*26*	57	*28*
Left colon	115	*33*	61	*32*	54	*34*	51	*35*	64	*31*
Right colon	142	*40*	81	*42*	61	*38*	56	*39*	86	*41*
**Tumor size (< 5 cm)**	174	*50*	94	*50*	80	*49.7*	71	*49*	103	*48*
**Differentiation**										
Well diff.	267	*76*	165	*86*	102	*65*	93	*65*	173	*82*
Poor diff.	84	*24*	27	*14*	57	*35*	51	*35*	36	*18*
**Lymphovascular invasion** (positive)	122	*35*	46	*24*	76	*48*	68	*47*	54	*26*
**Nervous invasion** (positive)	31	*1.0*	12	*6.0*	19	*12*	18	*12.5*	13	*6*
**Lymph nodes positive for CRC (**> 2**)**	42	*1.2*	1.0	*0.0*	41	*26*	35	*24*	7	*3*
**Colonic perforation** (yes)	13	*0.4*	8	*4.0*	5	*3.0*	4	*3.0*	9	*4.0*
**Tumor budding** (≥ 5 cells**)**	219	*62*	99	*52*	120	*75*	110	*79*	109	*53*
**Ulceration** (yes)	149	*42*	79	*41*	70	*44*	64	*44*	85	*41*
**Histological type**										
Adenocarcinoma	311	*89*	173	*90*	138	*87*	124	*86*	187	*90*
Mucinous/SRCC	40	*11*	19	*10*	21	*13*	20	*14*	20	*10*
**Pathological T stage**										
T1–2	80	*23*	65	*34*	15	*9*	14	*10*	63	*31*
T3–4	271	*77*	127	*66*	144	*91*	130	*90*	146	*69*
**Lymph node metastasis**										
No	207	*60*	189	*98*	18	*12*	–	*–*	–	*–*
Yes	144	*40*	3	*2*	141	*88*	–	*–*	–	*–*
**TNM stage**										
I + II	190	*54*	–	*–*	–	*–*	3	*2*	192	*92*
III + IV	161	*46*	–	*–*	–	*–*	141	*98*	17	*8*
**Schistosomiasis**	137	*39*	76	*40*	61	*38*	56	*39*	81	*40*

“–,” data is not applicable. *Abbreviation*: *N* number

### Survival analysis

The median follow-up time was 62.4 (1.25–134.4) months. During the follow-up, there were 41.6% (146 out of 351) patients who died. Mean and median time to OS was 62.54 and 62.85, respectively.

To investigate the association between schistosomiasis and clinical outcomes, we conducted K-M analysis according to schistosoma infection status. Result demonstrated that schistosoma infection was significantly associated with poor survival in total CRC patients (median survival time, 80.82 for CRC-S set and 119.20 for CRC-NS set; *P* = 0.0277) (Fig. 1b).

**Fig. 2:**
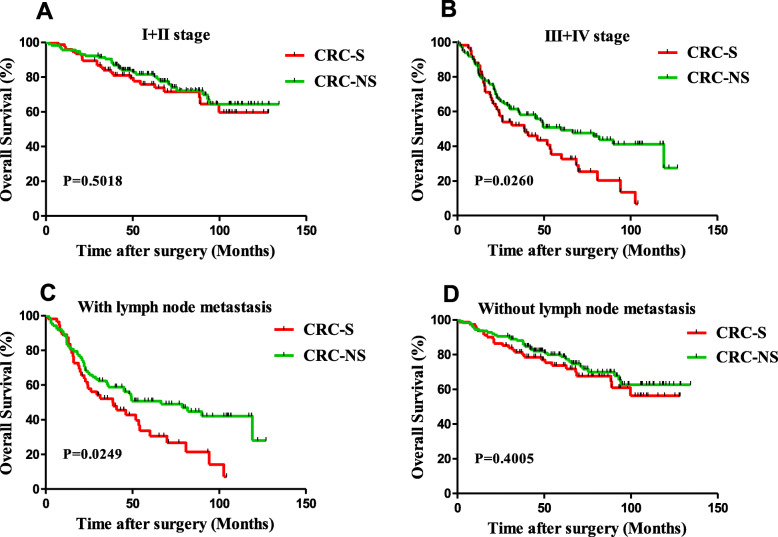
K-M analysis of OS in stratified CRC. **a** Patients with stage I–II tumors (*N* = 192, *P* = 0.5018). **b** Patients with stage III–IV tumors (*N* = 159, *P* = 0.0260). **c** Patients with lymph node metastasis (*N* = 144, *P* = 0.0249). **d** Patients without lymph node metastasis (*N* = 207, *P* = 0.4005). *P* value was calculated by log-rank test

Further analysis was conducted to explore the effect of schistosoma infection on CRC patients with similar stage tumors. In stage I–II set (*N* = 192), a K-M curve was plotted and found that schistosoma infection (40%) was uncorrelated with survival (*P* = 0.5018) (Fig. 2a). Nevertheless, in stage III–IV set (*N* = 159), K-M analysis showed a significant correlation between schistosoma infection and OS (*P* = 0.0260) (Fig. 2b).

In patients with lymph node metastasis (*N* = 144), schistosoma infection was observed in 39% (56 out of 144) CRC patients and associated with poor survival (*P* = 0.0249) (Fig. 2c). In contrast, there was no statistically significant difference observed in OS between CRC-S and CRC-NS patients without lymph node metastasis (*P* = 0.4005) (Fig. 2d).

### Univariate and multivariate analysis

The Cox proportional hazards model was used to determine factors that may influence OS of CRC patients. In the whole cohort, by univariate analysis and multivariate analysis (Table 2), gender (*P* = 0.003), TNM stage (*P* < 0.001), schistosomiasis (*P* = 0.025), lymphovascular invasion (*P* = 0.030), and lymph nodes positive for CRC (*P* < 0.001) were significantly independent predictors. Schistosomiasis was statistically significantly associated with decreasing OS.

**Table 2:** Univariate and multivariate Cox regression of clinicopathological for overall survival

Variable	All patients		Patients with stage I–II disease		Patients with stage III–IV disease		Patients with LNM		Patients without LNM	
	*P*	HR (95% CI)	*P*	HR (95% CI)	*P*	HR (95% CI)	*P*	HR (95% CI)	*P*	HR (95% CI)
**Univariate analysis**										
Age (< 60 years)	0.010	1.759 (1.142–2.708)	0.009	3.413 (1.355–8.597)	0.244	1.343 (0.818–2.205)	0.116	1.533 (0.900–2.611)	0.024	0.424 (0.201–0.894)
Gender (male/female)	0.008	1.602 (1.129–2.271)	0.046	1.897 (1.010–3.564)	0.018	1.670 (1.093–2.553)	0.020	1.714 (1.089–2.697)	0.103	1.584 (0.912–2.752)
Tumor size (5 cm)	0.913	1.018 (0.728–1.400)	0.735	0.909 (0.523–1.580)	0.480	1.157 (0.772–1.735)	0.233	1.297 (0.846–1.987)	0.435	0.816 (0.491–1.358)
Tumor site										
Rectum		Refer		Refer		Refer		Refer	Refer	
Left colon	0.908	1.025 (0.676–1.553)	0.670	1.168 (0.572–2.385)	0.926	0.975 (0.572–1.663)	0.926	0.975 (0.572–1.663)	0.942	1.025 (0.527–1.995)
Right colon	0.464	0.859 (0.572–1.290)	0.860	0.939 (0.467–1.888)	0.249	0.728 (0.425–1.249)	0.249	0.728 (0.425–1.249)	0.967	1.013 (0.531–1.865)
Pathological T stage	< 0.001	2.591 (1.562–4.297)	0.633	1.158 (0.634–2.116)	0.007	6.803 (1.675–27.633)	0.010	6.323 (1.554–25.722)	0.275	1.385 (0.541–1.486)
Lymph node metastasis	< 0.001	2.802 (2.012–3.902)	0.041	4.410 (1.063–18.289)	0.558	0.828 (0.441–1.556)	–	–	–	–
TNM stage	< 0.001	3.197 (2.271–4.501)	–	–	–	–	0.827	0.855 (0.210–3.481)	< 0.001	4.275 (2.203–8.298)
Differentiation	< 0.001	1.889 (1.334–2.674)	0.833	1.084 (0.510–2.307)	0.019	1.083 (1.083–2.466)	0.126	1.407 (0.909–2.177)	0.074	1.742 (0.957–3.169)
Lymphovascular invasion	< 0.001	3.251 (1.987–5.318)	0.163	1.538 (0.840–2.815)	0.011	1.702 (1.132–2.559)	0.019	1.670 (1.088–2.561)	0.036	1.782(1.039-3.056)
Nervous invasion	0.140	1.497 (0.876–2.559)	0.281	1.759 (0.630–4.909)	0. 866	1.056 (0.562–1.985)	0.943	0.976 (0.503–1.893)	0.176	1.888 (0.752–4.741)
Lymph nodes positive for CRC	< 0.001	4.006 (2.686–5.973)	0.007	16.949 (2.188–131.284)	< 0.001	2.178 (1.410–3.366)	< 0.001	0.723 (1.479–3.693)	0.001	5.338 (1.907–14.943)
Colonic perforation	0.541	0.700 (0.223–2.198)	0.824	1.174 (0.285–4.829)	0.768	0.809 (0.198–3.303)	0.461	0.475 (0.066–3.428)	0.512	1.475 (0.462–4.711)
Tumor budding	< 0.001	2.028 (1.400–2.938)	0.043	1.812 (1.019–3.221)	0.163	1.423 (0.867–2.336)	0.234	0.723 (0.424–1.232)	0.023	1.849 (1.087–3.146)
Schistosomiasis	0.044	1.399 (1.009–1.940)	0.474	1.225 (0.703–2.132)	0.011	1.699 (1.128–2.560)	0.019	1.674 (1.087–2.577)	0.428	1.229 (0.738–2.049)
Ulceration	0.624	0.9205 (0.660–1.282)	0.362	0.766 (0.4313–1.360)	0.971	1.008 (0.670–1.514)	0.649	1.104 (0.721–1.690)	0.189	0.698 (0.408–1.194)
Histological type	0.921	1.025 (0.626–1.680)	0.797	0.886 (0.352–2.230)	0.870	0.952 (0.529–1.713)	0.708	0.889 (0.482–1.641)	0.902	0.948 (0.408–2.206)
**Multivariate analysis**										
Gender	0.003	1.676 (1.178–2.384)	–	–	0.030	1.601 (1.047–2.450)	0.026	1.675 (1.064–2.639)	–	–
Pathological T stage	–	–	–	–	0.012	6.042 (1.476–24.729)	0.025	5.040 (1.228–20.678)	–	–
TNM stage	< 0.001	0.389 (0.267–0.567)	–	–	–	–	–	–	< 0.001	4.507 (2.303–8.818)
Lymph node metastasis	–	–								
Tumor budding	–	–							0.014	0.513 (0.301–0.876)
Differentiation	–	–	–	–	0.016	1.677 (1.101–2.555)				
Schistosomiasis	0.025	1.458 (1.049–2.027)	–	–	0.008	1.743 (1.153–2.635)	0.023	1.648 (1.070–2.538)	–	–
Lymphovascular invasion	0.030	1.461 (1.036–2.060)	–	–	–	–	–	–	–	–
Lymph nodes positive for CRC	< 0.001	2.256 (1.461–3.483)	0.007	16.8587 (2.176–130.580)	0.004	1.911 (1.230–2.969)	0.003	2.005 (1.267–3.175)	–	–

“–,” data is non-significant. *Abbreviation*: *CI* confidence interval, *HR* hazard ratio. *P* < 0.05 was defined as the criterion for variable deletion when performing backward stepwise selection

In late-stage (III–IV) CRC patients (Table 2), gender (*P* = 0.030), pathological T stage (*P* = 0.12), tumor differentiation (*P* = 0.016), schistosoma infection (*P =* 0.008), and lymph nodes positive for CRC (*P =* 0.004) were significantly independent prognostic factors for OS, while in early stage (I–II), lymph nodes positive for CRC (*P* = 0.007) was the only independent prognostic factor for OS in multivariate analysis.

In patients with lymph node metastasis (Table 2), gender (*P* = 0.026), pathological T stage (*P* = 0.025), schistosoma infection (*P* = 0.023), and lymph nodes positive for CRC (*P* = 0.003) were independent prognostic factors. In patients without lymph node metastasis (Table 2), TNM stage (*P* < 0.001) and tumor budding (*P* = 0.014) but not schistosoma infection were associated with OS in multivariate analysis. These results further proved that schistosoma infection may have different effects on CRC patients’ clinical outcomes, especially for patients with stage III–IV tumor and patients with lymph node metastasis.

### Association of schistosomiasis with clinicopathological features

The relationship between schistosomiasis and clinicopathological features is shown in Table 3. Patients with schistosomiasis were significantly older than the patients without schistosomiasis (median age 74.0 years vs 64.0 years, *P* < 0.001). The clinical stage of patients with and without schistosomiasis was similar (*P* = 0.816). In the total cohort, the male/female ratio was also higher in the CRC-S set (1.67 vs 1.43). Besides, in patients with lymph node metastasis, there were significant associations between male sex and female sex (*P* < 0.001). There were no significant differences in other clinicopathological characteristics between CRC-NS and CRC-S sets.

**Table 3:** The association between clinicopathological characteristics and schistosomiasis in CRC cohort

Characteristic		All patients		*P*	Stage I–II disease patients		*P*	Stage III–IV disease patients		*P*	With lymph node metastasis patients		*P*	Without lymph node metastasis patients		*P*
		CRC-NS (*N* = 214)	CRC-S (*N* = 137)		CRC-NS (*N* = 116)	CRC-S (*N* = 76)		CRC-NS (*N* = 98)	CRC-S (*N* = 61)		CRC-NS (*N* = 88)	CRC-S (*N* = 56)		CRC-NS (*N* = 126)	CRC-S (*N* = 81)	
**Age (< 60 years)**				< 0.001			< 0.001			< 0.001			< 0.001			< 0.001
< 60		78	5		41	5		37	0		34	0		44	5	
≥ 60		136	132		75	71		61	61		54	56		82	76	
**Gender**				0.467			0.695			0.520			< 0.001			0.588
Male		126	86		70	48		42	23		35	36		53	31	
Female		88	51		46	28		56	38		53	20		73	50	
**Tumor site**				0.274			0.750			0.829			0.806			0.030
Rectum		57	37		31	19		26	18		22	15		35	22	
Left colon		64	51		29	31		35	19		33	18		31	33	
Right colon		93	49		56	25		37	24		33	23		60	26	
**Tumor size**				0.985			0.597			0.581			0.372			0.443
< 5 cm		106	68		55	39		51	29		46	25		60	43	
≥ 5 cm		108	69		61	37		47	32		42	31		66	38	
**Differentiation**				0.570			0.577			0.700			0.952			0.417
Well diff.		165	102		101	64		64	38		57	36		108	66	
Poor diff.		49	35		15	12		34	23		31	20		18	15	
**Lymphovascular invasion**				0.909			0.732			1.000			1.000			1.000
Negative		138	90		87	59		51	32		46	30		92	60	
Positive		76	47		29	17		47	29		42	26		34	21	
**Nervous invasion**				1.000			0.766			1.000			1.000			1.000
Negative		194	125		108	72		86	54		78	49		117	76	
Positive		20	12		8	4		11	7		10	7		9	5	
**Lymph nodes positive for CRC**				0.867			0.998			0.710			1.000			1.000
≤ 2		189	120		115	76		74	44		67	42		122	78	
> 2		25	17		1	0		24	17		21	14		4	3	
**Colonic perforation**				0.966			0.482			0.373			0.299			0.487
No		206	132		110	74		96	58		87	53		119	79	
Yes		8	5		6	2		2	3		1	3		7	2	
**Tumor budding**				0.652			0.184			0.454			0.841			0.393
< 5 cells		83	49		61	32		22	17		20	14		63	35	
≥ 5 cells		131	88		55	44		76	44		68	42		63	46	
**Histological type**				0.731			0.470			0.590			1.000			0.633
Adenocarcinoma		191	120		106	67		85	53		76	48		115	72	
Mucinous/SRCC		23	17		10	9		13	8		12	8		11	9	
**Ulceration**				0.740			0.881			0.774			0.495			1.000
No		125	77		69	44		56	33		51	29		74	48	
Yes		89	60		47	32		42	28		37	27		52	33	
**Pathological T stage**				0.562			0.395			0.891			0.749			0.388
T1–2	I + II	51	29		42	23		9	6		8	6		43	23	
T3–4	III	163	108		74	53		89	55		80	50		83	58	
**Lymph node metastasis**				0.883			0.823			0.641						–
No		126	81		114	75		12	6		0	0		–	–	
Yes		88	56		2	1		86	55		88	56		–	–	
**TNM stage**				0.816			–			–			0.842			0.379
I + II		116	76		–	–		–	–		2	1		112	75	
III + IV		98	61		–	–		–	–		86	55		14	6	

“–,” data is not applicable. *Abbreviation*: *N* number. The association between schistosomiasis and clinicopathological characteristics was evaluated by using the chi-square and Fisher’s exact tests

In order to further investigate the effect of schistosomiasis on particular CRC population, we divided the whole cohort into different groups according to their clinical stage or the state of lymph node metastasis and further subgrouped them into CRC-S and CRC-NS set based on schistosomiasis. Except age, there were no correlations between other clinicopathological features and schistosomiasis when compared between CRC-NS and CRC-S sets in different groups (Table 3).

## Discussion

At present, there is sufficient evidence to conclude that *S. haematobium* has a role in causing some types of bladder cancer [21–23] and hepatocellular carcinoma [6, 10]. There is limited evidence to suggest that *S. japonicum* leads to CRC.

Our study demonstrated that schistosomiasis was an independently unfavorable factor for OS (*P* = 0.0260, Fig. 1b; *P* = 0.025, Table 2). These results indicated that schistosomiasis plays an important role in CRC progression and metastases. Shindo [24] reviewed 276 cases of large intestinal cancer with schistosomiasis and found significant differences between carcinoma with schistosomiasis and non-schistosomiasis-associated carcinoma in symptoms, age, sex, and histological findings, suggesting that schistosomiasis could induce the carcinoma. Ye et al. [25] reported that intestinal schistosomiasis was a risk factor for CRC and that the lesions caused by the disease might be considered precancerous. Liu et al. [26] reported that the history of colon schistosomiasis was a probable risk factor for the development of colorectal neoplasia, but only a few studies reported the clinicopathological characteristics and prognosis of patients with schistosomal CRC. This might be explained as follows. Firstly, there is little relevant clinical data in the medical literature, limited to case reports; physicians know little about it [27, 28]. Secondly, cases of colonic schistosomiasis are rare leading to a small sample size and potential bias in data analysis. Previous reports [29, 30] showed that the development of CRC-S occurs in a younger age group unlike our findings. This might be explained by the following reasons. First, since effective prevention and control measures were taken in China in 1983, the infection rate has decreased, which result in large quantity and relatively younger CRC-NS patients. Second, this disparity may be related to differences in hereditary factors and environmental carcinogens. Our results showed that there is also a male predominance (61%, Table 1) in the cohort, although there was no significant difference between CRC-NS and CRC-S patients (Table 3). The Qingpu District of Shanghai was previously predominantly rural and, as more males were engaged in farm work, is likely to be at greater risk for exposure [31–33].

In the cohort, there were 22 (1.7%) patients who have stage IV tumors, and the survival time ranged from 1.25 to 118 months. Although it is well known that stage IV tumors have a poor prognosis, we want to investigate the impact of schistosoma on CRC in the complete process.

Schistosomiasis was statistically significant for OS in the univariate analysis and was an independent prognosis factor in multivariate analysis in the whole cohort (*P* = 0.025, HR = 1.458, 95% CI = 1.049–2.027). When patients were stratified based on clinical stage or state of lymph node metastasis, schistosomiasis was also a significantly independent predictor, except in patients with stage I–II tumor or without lymph node metastasis. Therefore, our observation indicates that schistosomiasis may be a considerable risk for patients in different clinical stages, especially in the late clinical stage. This conclusion may increase the debate that schistosomiasis is a weak risk of CRC [5, 34, 35].

Our study has several limitations. First, because it was performed at a single institution, the uniformity of the results may be low. Further work will be needed to validate the present results. Second, patient selection bias is a possibility due to the nature of the retrospective study. Third, although we found a negative correlation between schistosomiasis and CRC outcomes, the precise functional roles of schistosomiasis in CRC progression and its underlying molecular mechanisms remain obscure. Chen et al. observed a variable degree of colonic epithelial dysplasia in 60% of cases with *S. japonicum* colitis and regarded these changes as a transition on the way towards cancer development in schistosomal colonic disease [36]. A similar conclusion was drawn by Yu et al. from their studies on different types of schistosomal egg polyps [34]. All these results suggested the pro-tumor mechanisms of *S. japonicum* in tumor tissues. Therefore, further analysis about the functional roles of schistosoma infection and underlying molecular mechanisms needs to be investigated. In addition, we were not sure if any of these patients suffered from familial cancer syndromes, such as Lynch syndrome. It was known that the proportion of patients with familial polyps and hereditary non-polyposis CRC syndrome is higher in young patients (≤ 40 years old) [37, 38]. In our cohort, there were seven patients (0.02%) under 40 years old. However, work will continue to examine this possibility. Lastly, it was reported that schistosomiasis results from the host’s immune response to schistosome eggs and the granulomatous reaction evoked by the antigens they secrete [39], and the process of granuloma formation will be accompanied by chronic inflammatio n[40, 41], which may induce the development of tumor. However, we could not provide evidence in this study and detection of inflammatory markers will be conducted to strengthen the hypothesis in further work.

In summary, our observations support the pathogenetic role of schistosomiasis and shed light on the adverse effects of schistosomiasis on CRC patients.

## Data Availability

The datasets used and/or analyzed during the current study are available from the corresponding authors on reasonable request.

## Abbreviations

AJCC: American Joint Committee on Cancer
CRC: Colorectal cancer
CRC-NS: Patients without schistosomiasis
CRC-S: Colorectal cancer patients with schistosomiasis
FFPE: Formalin-fixed paraffin-embedded
IMA: Inferior mesenteric artery
K-M: Kaplan-Meier
OS: Overall survival
*S. japonicum*: *Schistosoma japonicum*
